# Supplementary material for: The Drosophila circadian clock gene cycle controls the development of clock neurons
Source: PLoS Genet. 2024 Oct 21;20(10):e1011441. doi: 10.1371/journal.pgen.1011441 (PMC11527286; doi:10.1371/journal.pgen.1011441)
Supplement: S1 Fig — (A-B) The cyc01 mutant has disrupted sLNv morphology. (A) Representative confocal images of anti-PDF staining in Canton-S control and cyc01 adult male brains. The sLNvs and optic tract (OT) are indicated. Scale bar = 25 μm. The inserts on the right show the sLNv projections with the signal intensity adjusted for visibility in the cyc01 mutants. The top insert shows the distal (dorsal) area and the bottom insert shows the proximal (ventral) area of the sLNv projections. Scale bar = 10 μm. (B) Representative images of eight brains of; Pdf-RFP;cyc01 experimental flies stained with anti-RFP (magenta). Flies were raised at 28°C. Most of cyc01 mutant flies (~78.5%, 11 out of 14 brains) exhibit severe phenotypes in their sLNv morphology compared to the effects of cyc downregulation in Pdf+ neurons. (C-E) Representative confocal images of adult (C, E) and L3 larvae (D) control brains stained with anti-RFP (magenta). (C) To determine the degree of defasciculation of the sLNv ventral projections in adult brains, 6 concentric circles separated by is 25 μm were centered at the point of intersection (POI), where the projections of the sLNvs and those of the lLNvs intersect. The most distant circle does not reach the main branching point (BP) in control brains; therefore, the dorsal termini are not included. The number of intersections between either the sLNvs or the lLNvs and each concentric circle were quantified. (D) Dorsal projection branching in the larval sLNvs was measured by counting the number of intersections the sLNvs had at each of the 6 concentric circles separated by 12.5 μm. (E) Adult sLNv dorsal projection branching was measured by counting the number of intersections the sLNvs had at each of 8 concentric circles separated by 12.5 μm. This was adapted from a previous study [27] to capture the hyperextended projection phenotype of Pdf > Δ-Clk flies. (F-I) Quantification of the LNv morphology phenotypes of experimental flies in which a cycRNAi transgene was driven b [file pgen.1011441.s001.pdf]

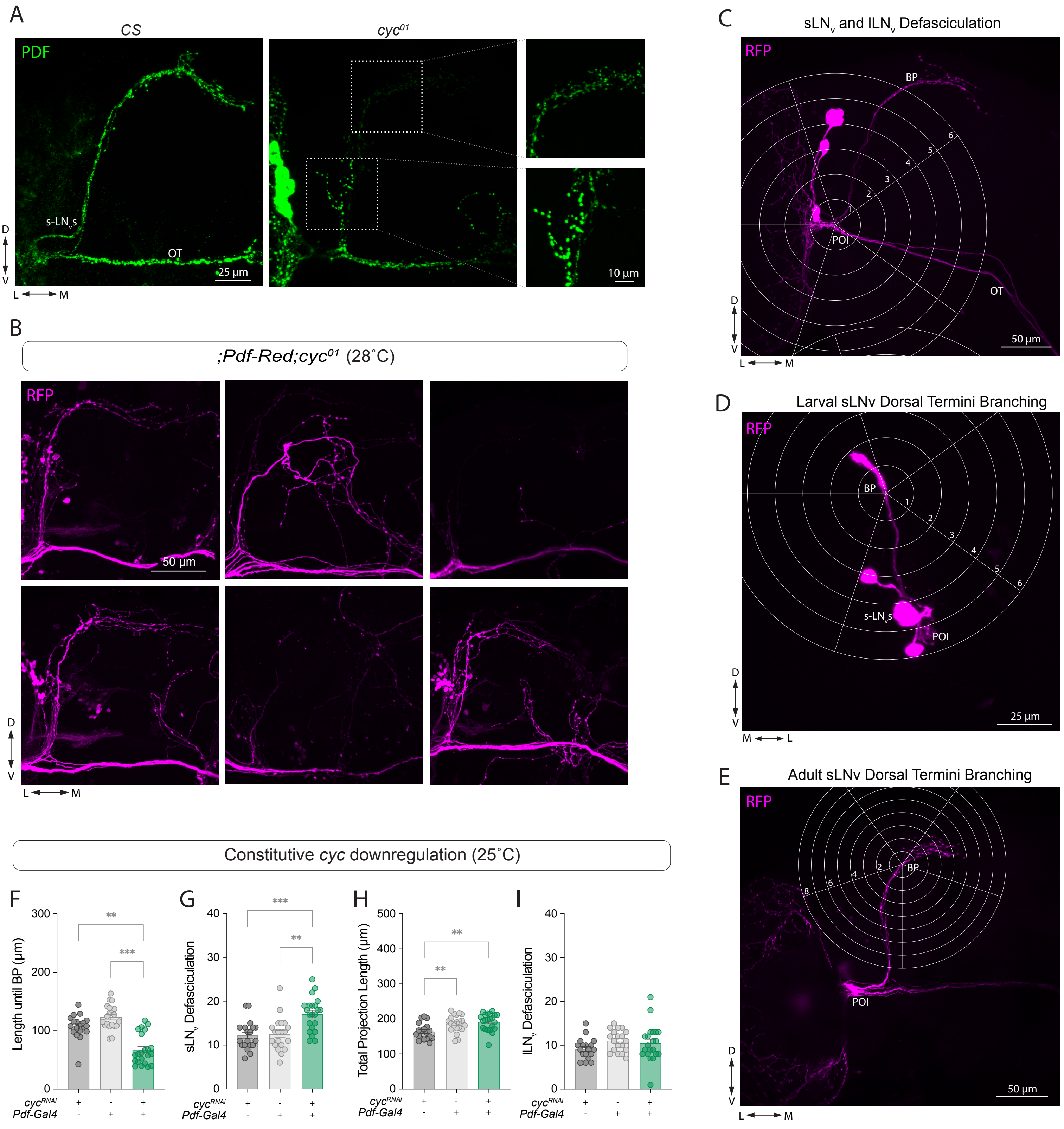
A
CS
cyc01
PDF
s-LNvs
OT
25 µm
10 µm
D
V
L
M
B
;Pdf-Red;cyc01 (28˚C)
RFP
50 µm
C
sLNv and lLNv Defasciculation
RFP
BP
POI
OT
1
2
3
4
5
6
50 µm
D
Larval sLNv Dorsal Termini Branching
RFP
BP
s-LNvs
POI
25 µm
E
Adult sLNv Dorsal Termini Branching
RFP
BP
POI
2
4
6
8
50 µm
Constitutive cyc downregulation (25˚C)
F
Length until BP (µm)
G
sLNv Defasciculation
H
Total Projection Length (µm)
I
lLNv Defasciculation
cycRNAi + - +
Pdf-Gal4 - + +
**
***
