# Supplementary figures and images for: The Drosophila circadian clock gene cycle controls the development of clock neurons
Source: PLoS Genet. 2024 Oct 21;20(10):e1011441. doi: 10.1371/journal.pgen.1011441 (PMC11527286; doi:10.1371/journal.pgen.1011441)

Constitutive  $\Delta$ -cyc Expression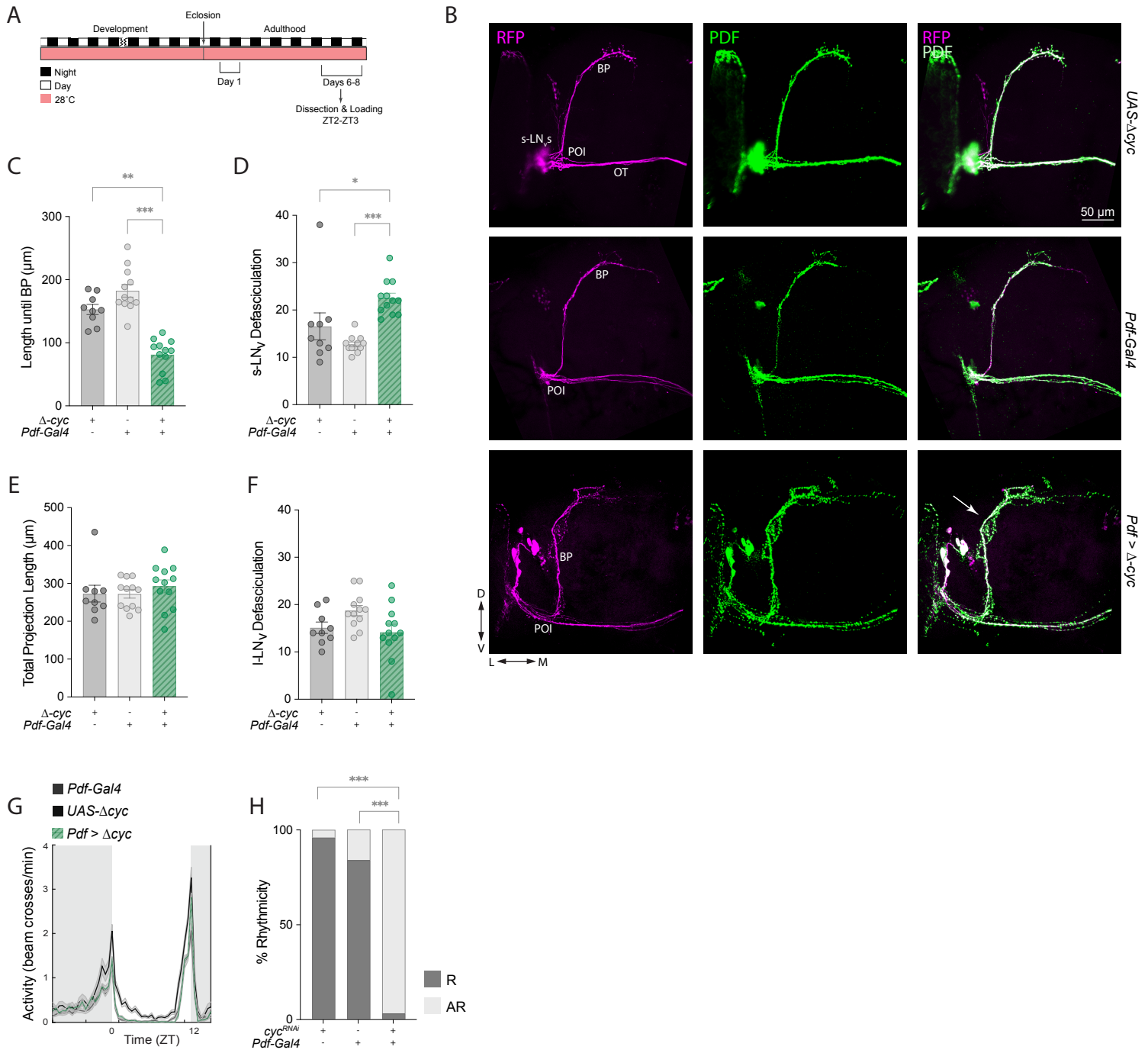

Supplement: S2 Fig — (A) Representative timeline of the experiments in the figure. Flies were kept in LD conditions at 28°C for their entire lifespan. Dissections were performed within days 6–8 post-eclosion at ZT2-3. (B) Representative brain confocal images of anti-PDF (green) and anti-RFP (magenta) staining in the sLNvs of flies in which Δ-cyc was constitutively expressed in the Pdf+ cells using a Pdf-Gal4;tub-Gal80ts driver. Each line also included a Pdf-RFP transgene. White arrows indicate branching of sLNv dorsal projections (left), and dorsal termini of the sLNv projections (right) for the experimental genotype. The images are representative of two independent experiments. Scale bar = 50 μm. LNv morphology was quantified by comparing the sLNv projection length until the branching point (C), the total number of intersections of the sLNv ventral projections (D), the full sLNv projection length (E), and the total number of lLNv intersections (F). One-way ANOVA was used to analyze normally distributed data (C, F). For nonparametric data sets, a Kruskal-Wallis tests followed by Dunn’s multiple comparisons tests was used (D,E). * p < 0.05, ** p < 0.01, *** p < 0.001. Error bars indicate SEM. Each dot corresponds to one brain. For each genotype: 9 ≤ n ≤ 12. (G-H) Behavioral phenotypes of constitutive Δ-cyc expression. Experiments were conducted at 28°C. (G) Population Activity (left) plots for flies during days 3–5 of the LD cycle at 28°C (see Table 1 for additional quantifications). (H) Percent rhythmicity for the indicated genotypes under DD. R = Rhythmic and AR = arrhythmic. Fisher’s exact contingency tests were used to analyze the percentage of rhythmic flies under DD (DD1-8). *** p < 0.001. Error bars indicate SEM. For each genotype: 24 ≤ n ≤ 32. (PDF) [file pgen.1011441.s002.pdf]

Adult-specific *cyc* downregulation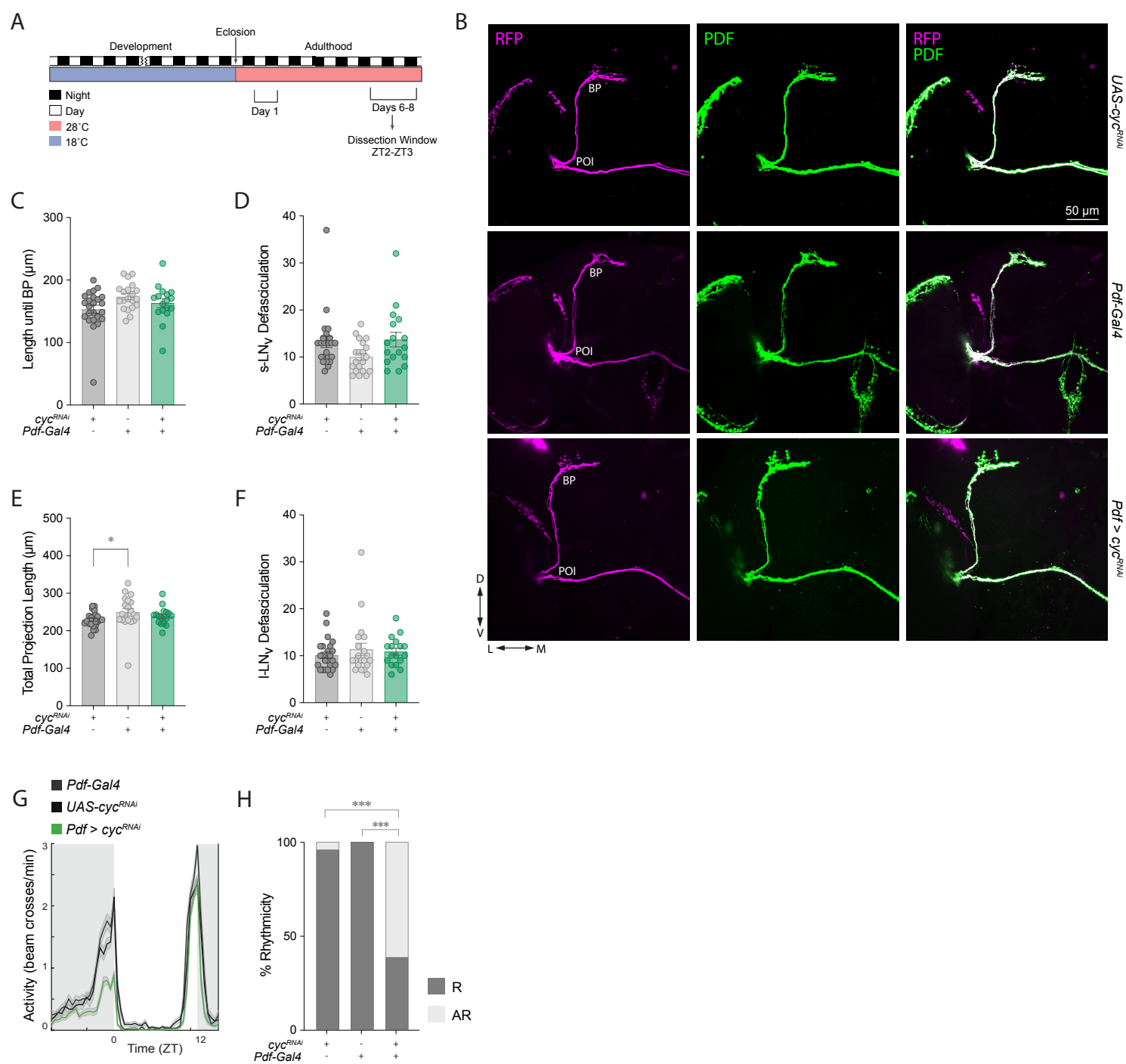

Supplement: S3 Fig — (A) Representative timeline of the experiments in the figure. Flies were raised in LD at 18°C, and transferred to 28°C immediately after eclosion. Dissections were then performed within days 6–8 post-eclosion at ZT2-3. (B) Representative confocal images of anti-PDF (green) and anti-RFP (magenta) staining in the sLNvs when cyc was downregulated exclusively after eclosion using a Pdf-Gal4;tub-Gal80ts driver. The images are representative of two independent experiments. Scale bar = 50 μm. Each line also included a Pdf-RFP transgene. Kruskal-Wallis tests followed by Dunn’s multiple comparisons tests were used to quantify the projection length until BP (C), the total number of intersections of the sLNv ventral projections (D), the full sLNv projection length (E), and the total number of lLNv intersections (F). * p < 0.05. Datasets are nonparametric (C-F). Each dot corresponds to one brain. For each genotype: 17 ≤ n ≤ 24. (G-H) Behavioral phenotypes of adult-specific cyc knockdown. Flies were raised in LD at 18°C, before being transferred to 28°C upon eclosion. Experiments were conducted at 28°C. (G) Population Activity plots for flies during days 3–5 of the LD cycle at 18°C (see Table 1 for additional quantifications). (H) Percent rhythmicity for the indicated genotypes under DD. Fisher’s exact contingency tests were used to analyze the percentage of rhythmic flies under DD (DD1-8). *** p < 0.001. Error bars indicate SEM. For each genotype: 25 ≤ n ≤ 31. (PDF) [file pgen.1011441.s003.pdf]

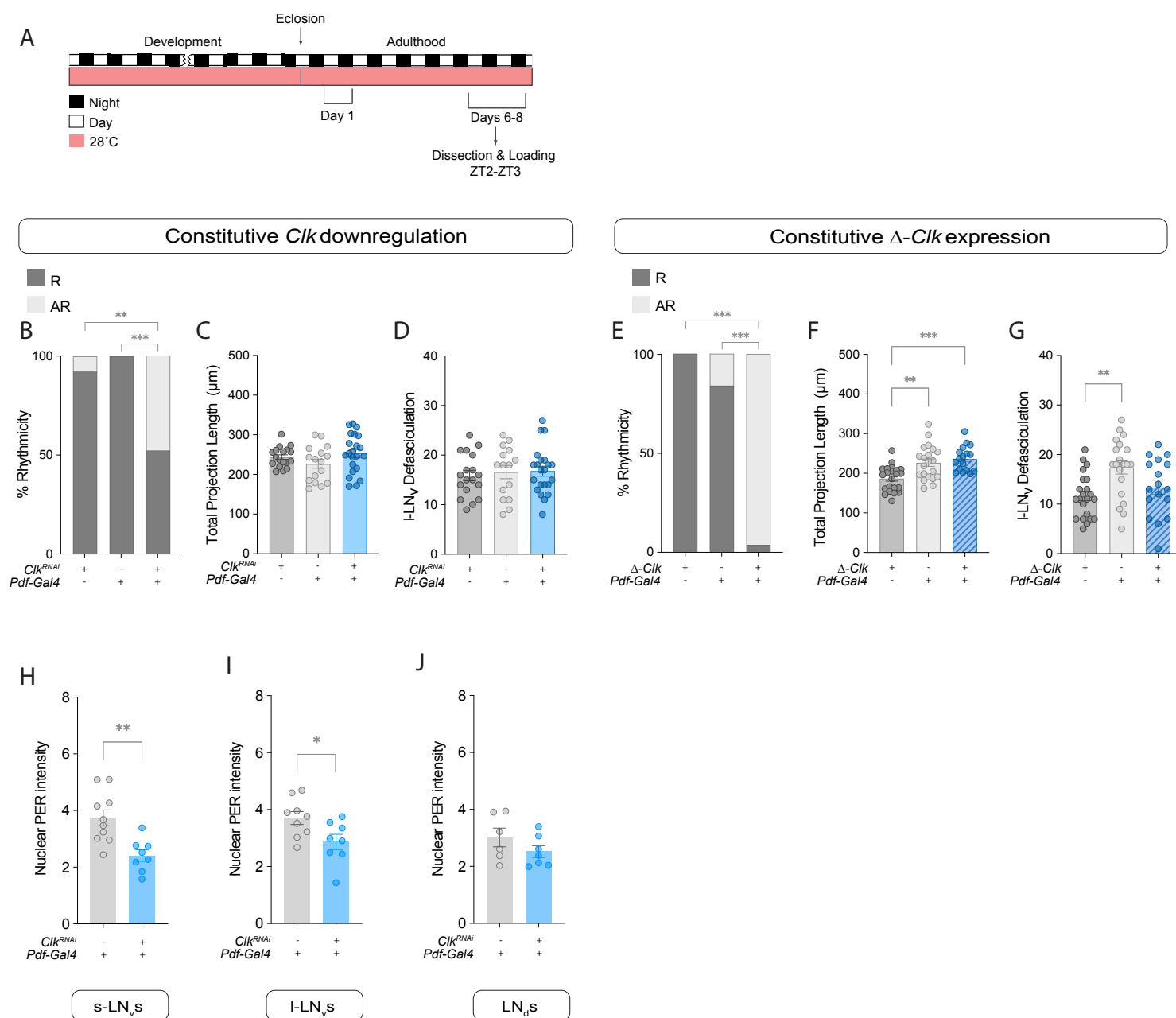

Supplement: S4 Fig — (A) Representative timeline. Flies were raised in LD at 28°C for their entire lifespan. Behavioral assays and dissections were performed within days 6–8 post-eclosion at ZT2-3. Experiments were conducted at 28°C. (B) Fisher’s tests were used to compare the percent of rhythmic flies of each indicated genotype (additional quantifications can be found in Table 1). ** P < 0.01, *** P < 0.001. For each genotype: 21 ≤ n ≤ 26. (C-D) Additional quantifications of effects of ClkRNAi expression in the Pdf+ cells in adult brains. Neither the sLNv total projection length (C) nor the lLNv projections (D) were affected. Datasets were quantified with ordinary one-way ANOVA tests followed by Tukey’s Multiple Comparisons tests. For each genotype: 16 ≤ n ≤ 22. (E) Fisher’s tests were used to compare the percent of rhythmic flies of each indicated genotype (additional quantifications shown in Table 1). For each genotype: 27 ≤ n ≤ 31. (F-G) Effects of Δ-Clk expression in the Pdf+ cells in adult brains. The sLNv total projection length (F) and the lLNv projections (G) were quantified using one-way ANOVA tests followed by Tukey’s Multiple Comparisons tests. Each dot corresponds to one brain. For each genotype: 17 ≤ n ≤ 22. (H-J) Mann-Whitney tests were used to compare nuclear PER intensity levels in the sLNvs (H), lLNvs (J), and LNds (J) in flies of the indicated genotypes. Flies were raised at constant 28°C for their entire lifespan and dissections were performed at ZT2-3 * p < 0.05, ** P < 0.01, *** P < 0.001. Error bars indicate SEM. (PDF) [file pgen.1011441.s004.pdf]

Larval *Clk* Downregulation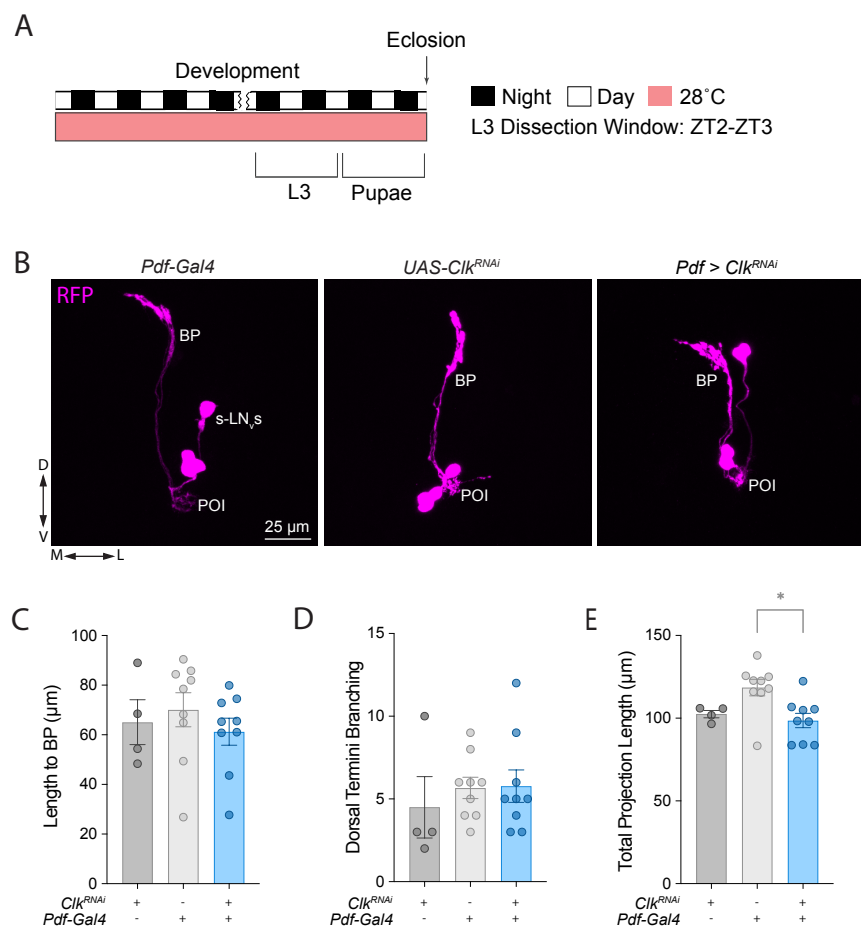

Supplement: S5 Fig — (A) Representative timeline of the experiments in the figure. Larvae were raised in LD at 28°C. Third instar larvae (L3) were dissected at ZT2-3. (B) Representative confocal images of anti-RFP (magenta) staining in the sLNvs when ClkRNAi was expressed in L3 larvae. Each line also contains a Pdf-RFP transgene. Scale bar = 25 μm. Kruskal-Wallis tests followed by Dunn’s multiple comparisons tests were used to compare the projection length from the POI to the BP (C), the total number of axonal intersections (D), and the total projection length from the POI (E). * p < 0.05. Error bars indicate SEM. Each dot corresponds to one brain. For each genotype: 4 ≤ n ≤ 9. (PDF) [file pgen.1011441.s005.pdf]
