# Supplementary material for: The Drosophila circadian clock gene cycle controls the development of clock neurons
Source: PLoS Genet. 2024 Oct 21;20(10):e1011441. doi: 10.1371/journal.pgen.1011441 (PMC11527286; doi:10.1371/journal.pgen.1011441)
Supplement: Attachment — Submitted filename: Cover Letter- Response to reviewers_23Sept2024.docx [file pgen.1011441.s008.docx]

September 23, 2024

PLoS Genetics

Editorial Office

Dear Dr. Copenhaver and Dr. Ewer,

On behalf of my co-authors, I am pleased to resubmit our manuscript, titled "The Drosophila Circadian Clock Gene Cycle Controls the Development of Clock Neurons."

Please find below a list of our responses to the reviewer’s comments. All changes made to the manuscript in response to specific comments are highlighted in blue in the corresponding sections. Additionally, as suggested by Reviewer #2 in their final comments, we have corrected the actograms in Figure 3G. We have also uploaded two versions of the manuscript: one with all changes to the text and figure legends highlighted in red, and a final version (dated today) with those changes incorporated.

We found the comments from our three expert reviewers insightful, and we are happy to hear that we have addressed their concerns. We have added new experiments, provided necessary clarifications, and made changes in the discussion of the results. We thank the three reviewers for their careful assessment of our work, as we believe the revisions have significantly improved the study. We also thank the editorial office and the academic editor for their work.

***Reviewer #1:***

*The manuscript examines LNv neuronal morphology in the cyc mutant background during both developmental and adult stages. The authors assert that CYC is essential for proper LNv neuronal morphology during development, in addition to its previously reported role in the molecular clock and claimed this is CYC’s non-circadian role. This point has been already brought up in previous reports (Ref# 23) but authors strengthen the point in this manuscript by scrutinizing the anatomical phenotype. However, further clarification and elaboration are needed, particularly regarding the anatomical phenotype of defective neurites in cyc mutant flies, identified as a fasciculation/de-fasciculation phenotype, which constitutes the primary focus of the manuscript (Main point #1). Additionally, it is somewhat unsatisfactory that the manuscript remains descriptive of the phenomenon and lacks explanation of the underlying mechanism. It is suggested to include the mechanistic explanation of how cyc might affect neurite fasciculation in the discussion.*

We thank the reviewer for carefully reading the manuscript and providing feedback. We agree that further clarification of the *cyc*^01^ anatomical phenotypes is important, and we have added this to the results section (see response to point #3 below) with examples of different *cyc*^01^mutants (new Fig S1B).

We have added the following paragraph to the discussion, which includes a discussion of how loss of cyc might affect neurite morphology (lines 336-350): “CYC/CLK may regulate neuronal fasciculation by modulating the expression of genes involved in cell migration or cytoskeletal dynamics. For example, increasing matrix metalloproteinases 1 (MMP1) expression reduces the complexity of the sLNv arborizations along the projections [31]. MMP1 promotes fasciculation in *Drosophila* motor neuron axons [56]. *Clk* has been shown to affect sLNv dorsal termini arborization through the activation of Mef2, which negative regulates Fas2 expression [52]. Our results from *Clk* downregulation show increased rather than decreased sLNv dorsal termini arborization. One important difference is that we used RFP to label the membrane rather than a PDF staining. As for *cyc*, among the sLNv morphology phenotypes reported in the literature, including those of other clock mutants, PDF/PDFR [57], and Rho GTPases [28], among others, the phenotype most similar to *cyc* downregulation is the downregulation of the Medea (Med). Med is homolog of the human tumor-suppressor gene DPC4 and is involved in the decapentaplegic (dpp) pathway [58], and its downregulation via RNAi in *Pdf*+ neurons results in decreased fasciculation along the projections of the sLNvs [57]. In addition, similar to *cyc* manipulations, developmental specific downregulation of Med leads to morphology phenotypes in adult clock neurons [57].”

*1. Figure 1: Given the critical role of the BP in the analysis, it is imperative to label the BP in every image, particularly in the mutant cases, similar to the labeling in Fig. 1C. Additionally, clarification is needed regarding the quantification of the degree of sLNv defasciculation. It remains unclear whether the authors refer to defasciculation of the axon bundle, dorsal termini, or both. The authors should elaborate on this aspect and provide a detailed explanation of how sLNv defasciculation was quantified.*

We have labeled the branching point (BP) in all relevant images and have expanded and clarified the methods section where the quantification of sLNv defasciculation is described (see 'Quantification of adult LNv morphology'). This section is now divided into separate paragraphs, each detailing a specific quantification, including all other LNv morphology quantifications. Graphical representations of the quantifications for ventral and dorsal projections are also provided in Supplementary Figure 1 (Fig. S1C and D, respectively).

2. *Experimental Conditions: All experiments, except for Fig. 1A-C, were conducted at 28°C. Considering the influence of temperature on developmental speed and its contribution to morphological defects, it is necessary to justify the choice of temperature and explain why experiments were not conducted at the standard temperature of 25°C. Additionally, cyc01 phenotype is referenced to all other phenotypes, cyc01 experiment has to be done at the same temperature as other experiments, 28°C.*

Our original experiments were conducted at 28°C to enable more direct comparisons with the adult-specific and development-specific downregulations of cyc, which involved shifting flies either from 18°C to 28°C or from 28°C to 18°C. Consequently, the constitutive downregulation (shown in Fig. 1E-H) was performed at 28°C, and the behavioral experiments presented in Table 1 were conducted at the same temperature for consistency. We have added this explanation to the results section (see lines 141-144) and the figure legend.

Following the reviewer's suggestion, we conducted the cyc^01^ experiment at 28°C. The results are similar to those seen at 25°C and are shown in the updated version of Figure 1 (Fig 1B-D).

*3. Pg5, line 129: The manuscript mentions that Cyc Ri flies resemble a minor version of the cyc01 phenotype without adequately describing the cyc01 phenotype categories. It is essential to distinguish between severe and minor phenotypes of cyc01 and provide the percentage distribution of these phenotypes.*

Most cyc^01^ mutant flies (~78.5 %, 11 out of 14 brains) exhibit severe phenotypes in their sLNv morphology compared to the effects of cyc downregulation in *Pdf*+ neurons. Additionally, these mutants displayed phenotypes not observed in *Pdf* > cyc^RNAi^ flies, such as pronounced misrouting or missing projections. The phenotypes of the cyc^01^ mutant flies were also much more variable than those of *Pdf* > cyc^RNAi^ flies. In order to clarify this, we now include examples of the diverse phenotypes observed in cyc^01^ flies in Supplementary Figure 1B and mentioned the percent of brains with severe phenotypes in the figure legend.

*4. Figure 3: Similar to Fig. 1, it is advisable to label the BP for other genotypes in Fig. 3. Clarification is needed regarding the white arrow. The authors should consider demonstrating the integrity of the molecular clock in the developmental stage KD condition to strengthen their argument regarding the molecular basis of arrhythmicity. Authors might show PER levels in the sLNV throughout the day.*

We have labeled the BP for all genotypes in Fig. 3, and simplified and clarified the meaning of the arrows in the figure legends.

We have included an experiment where we analyzed PER levels at two antiphasic time points (CT2 and CT14) on the second day of free-running (DD2) in flies in which cyc was downregulated only during development (shown in panel 3J). A description of this experiment has been added to the methods section. This analysis of PER subcellular localization revealed daily cycling, with nuclear PER levels higher at CT2.

*5. Figure 5: The authors should provide evidence of Clk knockdown in ClkRNAi flies, such as showing PER or CLK expression levels. The statement regarding the differential effects of Clk and Cyc downregulation on neuronal morphology may need to be tempered, considering the similarity between Clkdelta and cyc downregulation phenotypes (cyc0, cycRi, cycdelta), which might be attributed to less efficient Clk knockdown. This apply to the statement in the abstract, the title of the result section in pg8 and the many places in the manuscript.*

Following the reviewer’s suggestion, we have conducted a PER staining in *Pdf* > *Clk*^RNAi^ flies and found a significant reduction in the sLNvs and lLNvs. These results have been included in Supplementary Figure 4 (Fig S4H-J).

It is indeed possible that the *Clk* knockdown is less effective, and the milder behavioral effects observed with *Pdf* > *Clk*^RNAi^ compared to *Pdf* > *cyc* ^RNAi^ support this notion. We have modified the abstract and the title of the last results section and discussed the limitations of the tools in the discussion section (see lines 325-332). However, differential effects of *cyc* and *Clk* mutations have been previously described (Hendricks et al., 2023): cyc^01^ and *Clk*^jrk^ mutants showed differences in their sleep consolidation during the day and in their ability to recover after sleep deprivation. This has been included in the discussion as well (see lines 332-334).

*6. Figure 7F: The quantification of the distance from the OT appears somewhat arbitrary, and its significance is unclear. Instead, quantifying the intersection between dorsal projection and OT may provide a clearer explanation of the phenotype. Therefore It is suggested to denote intersection components in the axis title though.*

Following the reviewer's suggestion, we removed the quantification of the distance from the optic tract (OT). Previously, we had quantified the intersection between the dorsal sLNv projections and the lLN_v_s at the OT; we have updated the axis title to more clearly denote the intersection components as suggested (Fig. 7F). Additionally, we have provided a more detailed description of the quantification in the updated methods section titled “Quantification of Adult LNv Morphology.”

*7. Supplementary Figure 1: Similar to other figures, labeling the BP for every image is recommended. Additionally, the strong signal of concentric circles makes it difficult to visualize neurites, warranting consideration for improvement.*

We have now labeled the BP in Suppl. Fig. 1 and other relevant images. We have made the concentric circles in Fig S1 thinner to improve visibility. The quantification was conducted using 27’’ iMacs in which each image is shown in full view. The images shown in Suppl. Fig. 1 are simply to indicate the area of the neuron being quantified and the localization of the concentric circles relative to the POI and BP, but are several times smaller than the actual images used for analysis in the lab.

1*. Ensure proper citation for Fig. A in the text, and consider including a brief explanation of the experimental conditions.*

We have added the citation for Fig A in the text and included an explanation of the experimental conditions (see lines 115-116). Experimental conditions are also described in more detail in the figure legends.

*2. Correct the typo on page 8, line 250, Pdf>delta-cyc larvae.*

This typo has been corrected (line 263 in the new version of the manuscript).

*3. Pg. 13, line 370 if this reference 21 right?*

Thank you for detecting this mistake, reference #21 (Renn et al, 2000) was not correct and has been removed from the methods section. The correct reference was Scholl D.A.,1953.

***Reviewer #2****:*

*This manuscript shed light on the role played by the key circadian transcription factor CYC in the development of circadian pacemaker neurons. It also explores how its partner CLK contribute to the development of these neurons. Surprisingly, CLK and CYC downregulation or dominant-negative mutants result in different phenotypes, suggesting that CLK and CYC have distinct functions, and would thus not work only as heterodimers in clock neurons.

The study is very clearly presented, the results are solid and they extend significantly our understanding of the developmental role of CLK and CYC, which had been previously noted but not studied in detail. It would be interesting to clarify if CLK and CYC’s developmental roles are completely independent of each other. It would thus be interesting to manipulate both genes simultaneously, and see if the phenotypes become a combination of both mutants, or whether one set of phenotype caused by downregulation of one protein is dependent on the activity of the other protein.*

We thank the reviewer for this encouraging summary of our study and for the idea of the double knockdown.

The experiments shown in our manuscript involved a recombinant line with two transgenes on the second chromosome, w;Pdf-RFP;Pdf-Gal4; with or without a Tubulin-Gal80^ts^ transgene on the third chromosome, which was crossed to either uas-*cyc*^RNAi^ or uas-*Clk*^RNAi^ (or the dominant negative forms), both of which are also on the second chromosome. Therefore, to knockdown both genes simultaneously and still keep consistency with the other experiments shown throughout the study we would need to generate additional recombinant lines, and the process of generating such lines (which may or may not work) plus the experiments and imaging would take substantially longer than the allowed time for revision.

However, we agree that this is an important experiment and we are in the process of generating the recombinant lines needed to conduct this experiment. We are planning to pursue this in a future study, aimed at determining the extent to which *cyc* and *Clk* manipulations can affect neuronal development independently of each other.

*A few additional suggestions and questions:*

*1) Panel 3G: it seems the startle response occurs after the light-on transition. Is the LD shading on the actogram accurate?*

The LD shading was not accurate, panel 3G has been corrected

*2) Panel 3G and H. One panel 3G, experimental flies seem to be active randomly during the day, but on 3H they show clear evening anticipation, though reduced compared to control. Otherwise they show little activity during the day. Is the actogram on 3G really representative of the behavior of the experimental flies?*

We have replaced the actogram with a more representative actogram. The experimental flies are quite variable in their activity patterns outside the light- on and lights-off transitions, some have more activity throughout the day than others, but almost all show a clear anticipation of the evening transition.

3*) Flies with ablated LNvs usually show residual rhythmicity from non-PDF neurons. It seems that PDF-neuron specific downregulation shows a more severe phenotype, with no residual rhythmicity (panel 2H). Any speculation on why that is?*

This is a very interesting point. Evidence in the literature suggests that high levels of defasciculation lead to arrhythmicity, for example in Mef2 overexpression (Sivachenko et al., 2013). Conversely, manipulations increasing fasciculation or causing the loss of dorsal arborizations of the sLNvs typically do not significantly affect behavioral rhythms, for example when overexpressing Fas2 (Sivachenko et al., 2013) or via ectopic expression of Unc5 (Fernandez et al., 2020). It may be that increased fasciculation or hyperextension of the sLNv projections result in abnormal connections with other clock neurons or other downstream targets, or in inappropriate release of PDF in brain regions where it is not normally present.

*4) Line 310: I know what the authors meant to say, but this should be rephrased. CLK and CYC are expressed in many tissues, including glia, not just in clock neurons.*

We have removed that sentence, and we now better describe *cyc* expression in the last paragraph of the discussion.

*5) Line 311: Did single-cell RNA seq confirm the idea that cyc mRNAs are indeed present outside of the clock neurons? An alternative explanation would be that CLK works as a homodimer or as a heterodimer with an unknown partner to activate CYC expression and therefore the circadian transcriptional program in clock neurons, or in non-clock neurons when expressed ectopically. The present study indeed suggests CLK and CYC might be able to work independently of each other.*

Yes, single cell RNA seq revealed the presence of *cyc* mRNA in other neurons as well as outside the brain. These data can be found in a recent preprint from the Kadener lab at Brandeis, which we have added as a reference (Patop et al. 2023, Biorxv). The Fly Atlas database also revealed cyc mRNA outside the brain. Interestingly, several tissues show high *cyc* RNA levels but low *Clk* mRNA, and vice versa.

We have added the following sentences to the discussion (lines 357-362): “In addition, *cyc* mRNA was not enriched in the LNvs compared to other *elav*-expressing neurons in the head [61]. Single cell RNA sequencing data revealed that *cyc* mRNA is present in non-clock neurons as well as in various tissues throughout the fly’s body, with particularly high expression in the gut, ovaries, and testes [62]. In some instances, *cyc* mRNA expression levels are very high while *Clk* mRNA levels are low, such as in intestinal stem cells and the chordotonal organ [62].”

*6) Line 166. I think the % AR flies is actually 90%, according to Table I. Line 189: 77%*

This has been corrected (line 174).

***Reviewer #3****:*

*This study addresses an interesting question, namely the dual role of the core Drosophila circadian transcription factors CLK and CYC in cell-specification and development versus molecular oscillator function. Its conclusions are:
I) 'downregulating the clock gene cyc specifically in the Pdf-expressing neurons leads to decreased fasciculation both in larval and adult brains.'

II) 'This effect is due to a developmental role of cyc, as both knocking down cyc or expressing a dominant negative form of cyc exclusively during development lead to defasciculation phenotypes in adult clock neurons.'

III) 'Clk downregulation also leads to developmental effects on sLNv morphology, although cyc and Clk manipulations produce distinct phenotypes.'

IV)'Our results reveal a role for the circadian clock gene cyc in establishing the proper cellular morphology of the key clock pacemaker neurons, the sLNvs.'

The study's experiments are generally presented to a high standard that gives confidence in the technical execution of the assays.*

*The main points that require a response are:*

*1) Mapping of a neurodevelopmental phenotype of cyc loss-of-function phenotype to the PDF-expressing neurons is not perfectly new. A neuronal morphology defect for Clk and cyc mutants was first recognized by Park et al., PNAS 2000 and then shown to arise developmentally by Goda et al., PLoS Genetics 2011 with the latter study also demonstrating that rescue of abnormal morphology mapped to the PDF neurons. A deficit of these prior studies was that imaging was conducted at lower resolution and with anti-PDF antibodies rather than with the use of reporters whose expression was less dependent on CLK/CYC activity per se. Nevertheless PDF signal was not completely absent in cyc01 mutant flies and the residual signal (reported for 25-56% of adult brains) was sufficient to conclude that the dorsal PDF+ s-LNv projections had abnormalities. The statement in lines 101-102 is, therefore, exaggerated. And the discussion of the prior literature should be refined without pre-empting the need for and interest in the presented new data, which shows the actual extent of the morphological phenotype.*

Thank you for the encouraging comments on the technical standards of our work. We agree with this suggestion and have corrected the statement accordingly, and edited the discussion section where the study by Goda et al. is mentioned (lines 303-309).

“In *Clk*^jrk^ mutants, neither PDF nor *Pdf* mRNA can be detected in most larval [32] or adult sLN_v_s [23], and similar effects have been observed for the *cyc^02^* mutant [23]. However, around half of the *cyc*^01^ brains stained with PDF exhibit ‘stunted’ sLNv projections which appear to lack their dorsal termini [40]. This study by Goda et al. also showed that panneuronal rescue of *cyc* expression throughout development is sufficient to restore PDF expression in the LN_v_ dorsal projections of *cyc*^01^ mutants [40].”

*2) Both transgenic RNAi and dominant-negative mis-expression approaches come with technical limitations that are not addressed in the discussion. I appreciate that knockdown efficiency is not easy to assess in adult s-LNv and that PER is used as a proxy. Nevertheless, using a single RNAi line for cyc and Clk makes interpretation of the results vulnerable to any (off-target) side-effects. Moreover, using the over-expression of dominant negative alleles, while providing an independent approach, is not without its caveats either depending on the achieved level of over-expression and the emergence of non-native molecular interactions at higher concentrations. While I am happy for the results to be published I would request that the limitations of the chosen approach are acknowledged.*

We agree that it is important to address the limitations of the genetic tools employed, and we have therefore added the following sentences to the discussion section (lines 324-333): “Our results suggest that *Clk* and *cyc* manipulations produce different phenotypes, however, it is possible that this is partially due to a less effective knockdown of *Clk*. Behavioral experiments show that *cyc* knockdown in *Pdf*+ neurons result in a larger fraction of arrhythmic flies than knockdown of *Clk* (Table 1). Use of RNAi often reduces gene expression but does not completely eliminate it, and may lead to off-target side effects. In addition, RNAi efficiency may vary over time. Expression of dominant negative alleles was used as an independent approach, but this method has limitations as well: over-expression levels for *cyc* and *Clk* may differ, and non-native molecular interactions may occur at high concentrations. However, differential effects of *cyc* and *Clk* mutations have been previously described: cyc^01^ and *Clk*^jrk^ mutants showed differences in their sleep consolidation during the day and in their ability to recover after sleep deprivation [47].”

*3) I suspect that the authors agree that it is difficult to interpret the meaning of conclusion III. Given the technical limitations mentioned in point 2) above it may very well be that there is no reason to invoke novel partners or limited homodimerization for CLK and CYC in their neurodevelopmental role. The difference in phenotypes between Pdf>Δ-cyc and Pdf>Δ-Clk could reflect a difference in the native amounts of CLK versus CYC rather than a qualitative difference in the function of these transcription factors. Further, the behavioural phenotype of Pdf>ClkRNAi tubGal80ts at 28C in Table 1 is apparently milder than that for the comparable cycRNAi suggesting that knockdown efficiency and/or residual function may not have been the same. While the discussion conspicuously stays away from adressing this issues, I would prefer it if the question whether CLK and CYC act as an obligate heterodimer in their neurodevelopmental function was tackled, if only to formulate what future experiments might resolve this question.*

It is indeed possible that the *Clk* RNAi is less effective than the *cyc* RNAi, and at the moment we are not able to completely rule out the possibility that the different phenotypes are due to lower efficiency of the *Clk* knockdown. However, the morphological phenotype of *Clk* knockdown does not resemble a milder phenotype of *cyc* knockdown as the main differences are in different parts of the neuron: *cyc* in the ventral area and *Clk* in the dorsal area. This could still due to a more effective *cyc* manipulation having an effect earlier in development, when those projections begin to extend from the cell bodies.

We have added the following paragraph to the discussion (lines 362-367): “An interesting question for future studies is whether CLK and CYC act as an obligate heterodimer in their neurodevelopmental function and other possible non-circadian roles. In mammals, BMAL1 can dimerize with NPAS2 in addition to CLOCK [63], and a recent study in *Drosophila* detected co-binding of CYC and FOXO in the promoter region of *vrille* [64]. Single seq RNA sequencing in the sLNvs and other clock neurons, comparing the effects of *cyc* vs *Clk* downregulation, could help clarify the degree to which they function independently.”

*4) I note that experimental conditions generally refer to constant 28C. This raises the question whether PDF s-LNv development in the presence of Pdf>cycRNAi or Pdf>ClkRNAi or Pdf>Δ-cyc or Pdf>Δ-Clk would be more normal in the absence of tubGal80ts at 25C. I am not asking for more experiments (unless the data is already available), but it should be acknowledged that development occurs more quickly at 28C and there may, therefore, be less room for error in patterning neuronal morphology at 28C than at a lower temperature, leaving open the possibility that neurodevelopmental phenotypes arise in a somewhat temperature-dependent manner regardless of the impact of temperature on the transgenic gene expression.*

We agree that it is important to consider the effects of temperature on development and since the experiments could be completed within a reasonable timeline, we have conducted two new experiments to address this concern. We downregulated cyc in *Pdf*+ neurons in flies raised under LD conditions at constant 25°C and quantified the sLNv morphology phenotypes (Suppl. Fig 1F-I). Additionally, we conducted a standard LD-DD behavior experiment at 25°C, in flies raised at constant 25°C (Table 1, bottom section). The effects observed at 25°C were similar to those observed at 28°C, both in terms of neuronal morphology and behavior.

*Table 1 is corrupted typographically and lacks statistical analyses. This needs to be remedied.*

We have fixed the formatting issues of Table 1, and we included a new table (Table S1) in which all the details of the statistical analyses are included.

Thank you for considering our manuscript for publication in *PLoS Genetics.*

Sincerely,

María de la Paz Fernández

Asst. Professor of Biology

Biology Building

Lab: A504 / Office: A502

Indiana University

1001 East Third St.

Bloomington, IN 47405

812-855-9971

*fernanm@iu.edu*
